# Transcranial Ultrasound Innovations Ready for Broad Clinical Application

**DOI:** 10.1002/advs.202002026

**Published:** 2020-10-27

**Authors:** Roland Beisteiner, Andres M. Lozano

**Affiliations:** ^1^ Department of Neurology Medical University of Vienna Vienna 1090 Austria; ^2^ Division of Neurosurgery Department of Surgery University of Toronto Toronto ON M5T 2S8 Canada

**Keywords:** brain therapy, neuropsychiatric disease, ultrasound

## Abstract

Brain diseases are one of the most important problems in our rapidly ageing society. Currently, there are not many effective medications and surgical options are limited due to invasiveness and non‐invasive brain stimulation techniques cannot be well targeted and cannot access deep brain areas. A novel therapy is transcranial ultrasound which allows a variety of treatments without opening of the skull. Recent technological developments generated three revolutionary options including 1) targeted non‐invasive surgery, 2) highly targeted drug, antibody, or gene therapy via local opening of the blood–brain barrier, and 3) highly targeted brain stimulation to improve pathological brain functions. This progress report summarizes the current state of the art for clinical application and the results of recent patient investigations.

## Graphical abstract

Novel ultrasound techniques allow revolutionary new brain therapies. With pulsed ultrasound, highly targeted therapeutic brain stimulation to improve pathological brain functions is now possible. Higher energies allow targeted non‐invasive surgery and local opening of the blood–brain barrier for highly targeted drug, antibody, or gene therapy.

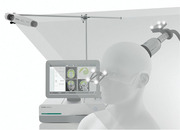

## 1 Introduction

Brain diseases are one of the most important problems in our rapidly ageing society. Currently there are not many effective medications, which is due at least in part to problems for novel drugs to reach the brain (blood–brain barrier). Application of surgery is limited due to invasiveness, particularly in the elderly. As a third therapeutic approach, support of restorative brain processes via brain stimulation has been tested. However, available electrophysiological techniques are limited regarding targeting and access to deep brain areas. Improvement for all three approaches is now possible via a novel development: transcranial ultrasound for the brain. Progress with this technology has been extremely rapid and devices for clinical application have been developed within less than a decade. The first therapeutic option with transcranial ultrasound is non‐invasive and highly targeted surgery. High intensity focused ultrasound (HIFU) beams are used to destruct pathologically active axons and neurons. The second therapeutic option is driving the clearance of pathological brain proteins or allowing the targeted delivery of drug, antibodies or gene therapy by focal and reversible opening of the blood–brain barrier. As a means of brain protection, the blood–brain barrier hinders the two‐way translocation of large intrinsic brain molecules and therapeutic agents in and out of the brain. Recent patient studies have shown that focal access to diseased brain tissue via focal blood–brain barrier opening is now possible. The third therapeutic option concerns non‐invasive and highly targeted brain stimulation without affecting the blood–brain barrier. This technique allows improvement of diseased brain functions via support of neuroplastic reorganization. All three options allow novel treatments of brain diseases without opening of the skull. For ultrasound surgery and neuromodulation meanwhile approved clinical systems exist. This progress report summarizes the current state of the art for clinical application and the results of recent patient investigations.

## 2 Ultrasound Surgery

**Figure 1:**
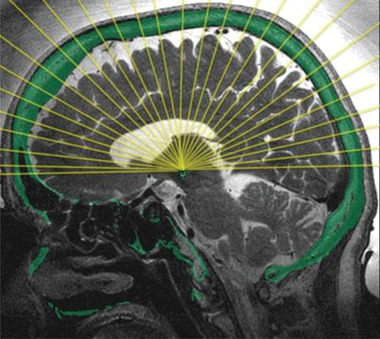
Principle of a helmet system with 1024 ultrasound transducer elements for focal surgery or focal blood–brain barrier (BBB) opening. Reproduced with permission. Copyright INSIGHTEC (www.insightec.com).

**Figure 2:**
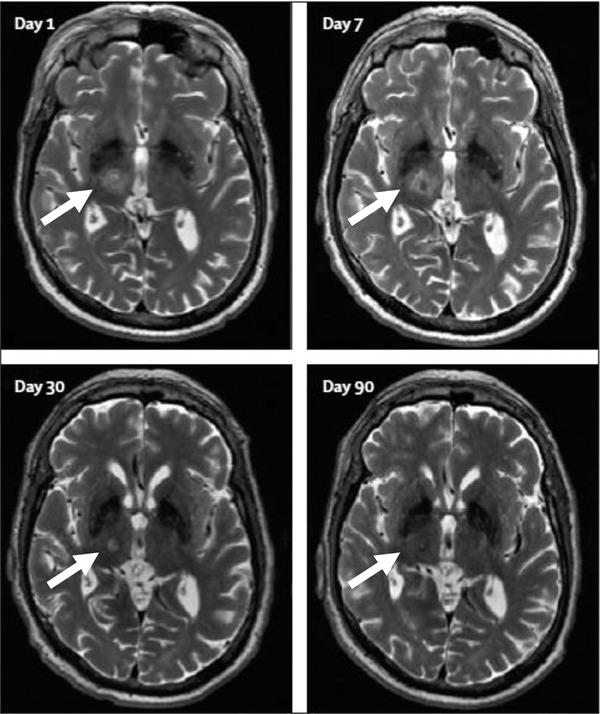
Ultrasound surgery of the right unilateral thalamus (white arrow) of a patient with essential tremor performed with a focused ultrasound helmet system (compare Figure 1). Lesion generation (day 1) and subsequent healing are shown over a period of 90 days. Adapted with permission.^[^ ^7^ ^]^ Copyright 2013, Elsevier.

There are a number of ultrasound‐based ablative techniques that could be used to make therapeutic brain lesions (reviewed in ref. ^[^ ^1^ ^]^) including cavitation enhanced ablation,^[^ ^2^ ^]^ histotripsy,^[^ ^3^ ^]^ interstitial ablation,^[^ ^4^ ^]^ and thermal based focused ultrasound ablation. Most clinical experience to date with cranial ultrasound surgery, has been performed with magnetic resonance imaging guidance thermal based focused ultrasound (MRgFUS) ablation. This technique uses multiple beams to produce a small and very focal high temperature locus inside the brain for tissue ablation (**Figures** **1** and **2**).^[^ ^5^, ^6^ ^]^ The technique is called HIFUS and is particularly promising for treating motor symptoms of various brain diseases. FDA approval for essential tremor exists since 2016 and for tremor dominant Parkinson's disease since 2018.

HIFUS for tissue ablation mostly applies midfrequency ultrasound systems (650 kHz) for high temperature thermal ablation. Alternatively, low‐frequency systems (220 kHz) for ablation via mechanical tissue destruction (histotripsy) would be possible, but harder to control. For thermal ablation, energies greater than 1000 W cm^−2^ generate DNA fragmentation, coagulative necrosis, and cellular death. For generating a highly focal surgery spot, a typical HIFUS system applies 1024 transducer elements within a helmet array, which is coupled to the skull via a water‐filled silicone rubber diaphragm, tightly placed around the patient's head. Circulating cooled degassed water (15–20 °C) avoids overheating of the bone.

Recent methodological advances concern refinement of ultrasound transducer technology and integration of individual imaging data like CT scans, MR scans, and diffusion tensor imaging scans, which are used for sophisticated targeting. This imaging information is important, since skull inhomogeneity has to be regarded, and common surgery targets (e.g., ventro‐intermediate nucleus of the thalamus, globus pallidus internus) are not visible on conventional anatomical MR scans. Electronic phase and amplitude control for each of the 1024 transducer elements ensures a precise in‐phase ultrasound convergence at the target with an accuracy below 2 mm. In addition, MR thermometry allows real‐time temperature monitoring to guide the therapy. Considerable progress has also been made to avoid unwanted cavitations (small vapor‐filled cavities induced by rapid changes of pressure in places where the pressure is relatively low) during HIFUS treatment. Such cavitations produce additional mechanical destructions which are hard to control. Recent efforts in HIFUS systems predict and detect cavitations.^[^ ^8^ ^]^

A typical HIFUS surgery procedure is performed in awake patients using 650 kHz and high temperature thermal ablation. First, transient clinical reactions are induced by escalating doses of low power sonication to a sublesion temperature of about 45 °C. This allows reversible testing of clinical efficacy and adverse effects at the target. Clinical monitoring is done via immediate clinical feedback, live thermography, and anatomical MR imaging. When the final target has been determined, several high‐power sonications are applied under the guidance of MR thermometry and with a focal temperature of 55–60 °C. This is accompanied by radiological evaluation of thermal lesioning location and clinical evaluation for safety. A unilateral surgery of the ventro‐intermediate nucleus of the thalamus may require 10 to 15 sonications with approximately a 2 h total treatment time.

Recent clinical studies demonstrate that the procedure is safe and ready for broad clinical application. Most clinical reports concern improvement of motor symptoms for diseases with movement related dysfunctions. New data indicate that HIFUS surgery represents a major benefit particularly for patients with medically resistant essential tremor.^[^ ^9^ ^]^ Essential tremor affects daily life tremendously, since controlled actions and muscle control are considerably affected. Recent follow up studies over 2–4 years post‐treatment show long‐term benefits for these patients.^[^ ^10^, ^11^, ^12^ ^]^ Typically, HIFUS is targeted to the ventro‐intermediate nucleus of the thalamus in one side of the brain (thalamotomy) and destruction of this malfunctioning area is guided by intraoperative symptom monitoring. 2–4 years post HIFUS treatment, patients’ hand tremor score was still improved by 38–56% and disability, action and postural scores by up to 75% compared to the pretreatment situation. Since the ultrasound technique is non‐invasive, a very recent publication reported applicability even for geriatric patients over 90 years of age.^[^ ^13^ ^]^ It is important to realize that these and other multimorbid patients cannot be treated by invasive surgery, and thus the novel ultrasound techniques are the only option to treat medically resistant essential tremor in this population. Another important disease with disabling tremor symptoms is multiple sclerosis (MS), which often affects very young people. Using the technique of HIFUS thalamotomy, a first successful treatment has recently been described by Máñez‐Miró et al.^[^ ^14^ ^]^ It is also younger people who are affected by a motor disease called focal hand dystonia. Here, patients experience uncontrollable muscular cramps which are related to a specific occupation—for example, musicians playing a musical instrument. Outside the specific occupational context, motor functions are normal, but occupation‐related symptoms often lead to a permanent occupational disability. Treatment is difficult, but invasive lesioning of the ventro‐oral nucleus of the thalamus may cause dramatic improvements. Horisawa et al.^[^ ^15^ ^]^ demonstrated therapeutic application of non‐invasive HIFUS also for focal dystonia. Targeting the ventro‐oral nucleus of the thalamus, they successfully treated a guitarist.

A major movement disorder is Parkinson's disease (PD) and several lines of evidence meanwhile exists that ultrasound surgery may also improve motor symptoms in PD. Transcranial ultrasound has been targeted to either the globus pallidus and pallidothalamic tracts or the subthalamic nucleus to improve the cardinal features of Parkinson's disease. A recent positron emission tomography (PET) study reported that the novel therapy reduces the abnormal metabolic brain pattern produced by PD. Even more importantly, this pattern normalization correlated with clinical improvement of the patients’ motor scores.^[^ ^16^ ^]^

Besides treatment of motor disorders, ultrasound surgery is also being examined in psychiatric diseases. Several studies investigated benefits for patients with obsessive–compulsive disorder (OCD). The target is a bilateral lesioning of the anterior limb of the internal capsule. A recent 2 year follow‐up in patients with treatment‐refractory OCD showed significant improvements in obsessive‐compulsive, depressive and anxiety symptoms.^[^ ^17^ ^]^ A frequent brain disease with cognitive symptoms is depression. In treatment‐resistant major depressive disorder, bilateral lesioning of the anterior internal capsule has been tried with various approaches. Recently, a first successful treatment with HIFUS ablation has been published with persisting improvements of depression scores during 1 year follow‐up.^[^ ^18^ ^]^

Overall, recent technical progress in transcranial ultrasound surgery allows successful novel treatments of a variety of brain diseases. Further methodological improvements are underway and concern target identification, handling of heterogeneous skull regions, phase‐correction algorithms, head stabilization techniques, definition of treatment end points and translation of the procedure for children. Since evaluation of sufficient lesioning currently depends on clinical testing, inclusion of real‐time feedback by real‐time functional MRI (fMRI) also seems promising. Clinically, the field is already rapidly expanding and treatments for additional diseases will soon be available. Promising clinical studies are currently running for epilepsy and neuropathic pain.^[^ ^19^ ^]^ Ablation of brain tumors has also been tried,^[^ ^20^, ^21^ ^]^ however, for this group of brain diseases the new technique of reversible blood–brain barrier opening may be an alternative strategy.

## 3 Ultrasound Blood–Brain Barrier Opening

A large number of potential clinical applications are also evident for the newly emerging technique of reversible and focal blood–brain barrier (BBB) opening with transcranial ultrasound (reviewed in ref. ^[^ ^22^ ^]^). Within the last years, tremendous progress has been made to allow transfer of drugs, antibodies, viral vectors and immune cells from the blood to a specific brain spot.^[^ ^23^ ^]^

**Figure 3:**
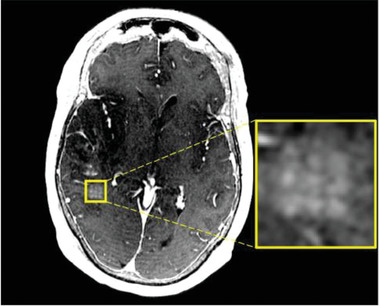
Blood–brain barrier opening of a larger brain area via sonication of a 3 × 3 grid consisting of 3 × 3 × 6 mm cubes.^[^ ^27^ ^]^ Extravasation of contrast agent from the blood to the brain is visible and corresponds to the precisely sonicated grid pattern (yellow square). Reproduced under the terms of the CC‐BY‐4.0 International license.^[^ ^27^ ^]^ Copyright 2019, Springer Nature.

Currently, four different types of transcranial ultrasound systems exist for reversible blood–brain barrier (BBB) opening: 1) external helmet systems with up to 1024 transducer channels (Figure 1), 2) external single‐channel devices, 3) implantable single‐channel devices, and 4) implantable multichannel devices. The principle technology of transcranial ultrasound transmission is similar to HIFUS systems for ultrasound surgery. However, ultrasound frequencies and applied energy levels are much lower, and the method is therefore called low intensity focused ultrasound (LIFUS). With external helmet systems 220 kHz frequencies and 4–5 W energy are typically used and the size of the ultrasound focus is about 3 mm in diameter. To open a larger than 3 mm target area, targets may be divided into cubic grids:, for example, a 9 × 9 × 6 mm^3^ target area is divided in nine 3 × 3 × 6 mm cubes which are successively sonicated, requiring about 50 s for a single sonication of the whole target area (**Figure** **3**).^[^ ^24^ ^]^ For achieving effective local BBB disruption without cell damage, injection of microbubble contrast agents (1–10 µm lipid spheres encapsulating a perfluorocarbon gas) are required, which are already in longstanding clinical use. Expansion and contraction of the injected microbubbles at the ultrasound focus result in stretching of the local capillary vessel walls and effect their endothelial cells in various ways.^[^ ^25^ ^]^ Major mechanisms suggested for BBB opening include 1) opening of the tight junctions between neighboring endothelial cells, 2) activation of transcellular substance transport through the endothelial cells via small vesicles, and 3) formation of open channels through endothelial cells via merging of several vesicles. This allows delivery of small drug molecules and chemotherapeutic agents, monoclonal antibodies, enzymes, neurotrophic factors, DNA and viral vectors for gene therapy, immune cells (natural killer cells for tumors) or neuronal stem cells and endogenous immunoglobulins (IgG and IgM) to the brain (reviewed in refs. ^[^ ^5^, ^22^ ^]^). To further support delivery of these items, use of liposomes, nanoparticles, drug‐loaded microbubbles, or magnetic attraction of cells has been suggested. An additional mechanism which supports tissue accumulation of substances at the ultrasound focus is an inhibition of efflux transporters which transport substances back to the blood.^[^ ^26^ ^]^ The extent of BBB opening depends on acoustic parameters (acoustic pressure, frequency, burst length) and microbubble size/microbubble concentration. The opening is reversible and can be fully closed 6–24 h after sonication.^[^ ^24^ ^]^

Similar to HIFUS, recent methodological advances concern transducer technology and integration of individual imaging data for precise targeting. In addition, development of reliable monitoring systems for inertial cavitation has been mandatory to avoid mechanical cell destruction. Inertial cavitation means a violent collapse of microbubbles due to inertia of the surrounding medium, resulting in jet streams, shock waves, and extremely high local temperatures. For every patient and target area, the specific cavitation threshold is determined. This is the energy level at which system hydrophones detect a subharmonic acoustic feedback from the target, which indicates occurrence of inertial cavitation. Actual BBB opening is then performed at 50% of this cavitation threshold.

A typical clinical BBB opening procedure with a helmet system can exactly target deep and variable areas of the brain up to about 4 cm^3^. Difficulties exist for superficial and larger targets, which may be better handled with other devices. With helmet systems, targeting requires fixation of a stereotactic frame to the patient's head and the transducer helmet. Head shaving is also required. BBB opening is then performed inside an MR system with the patient awake and supine. Based on preplanning images, the helmet system targets the treatment area. Then microbubble contrast agent is given intravenously, followed by LIFUS to the target spots. Local brain temperature is monitored via MR thermometry and inertial cavitation is avoided by analysis of acoustic feedback. Typically, one complete target area is sonicated 2–8 times, and after each sonication the patient is questioned for adverse events and may be examined. The whole procedure lasts 2–4 h.

Recently published first clinical studies indicate a broad range of applications. For innovative treatment of brain tumors, a pioneering study was performed in 15 glioblastoma patients sonicated with a single channel device implanted within the skull bone overlying the tumor area. Such devices are being commercially developed.^[^ ^28^ ^]^ Pressure levels up to 1.1 MPa were safe and well‐tolerated by all patients.^[^ ^29^ ^]^ The first tumor study using a helmet system was recently published by Mainprize et al.^[^ ^27^ ^]^ 1 h before BBB opening, 5 high‐grade glioma patients received a dose of chemotherapy. Successful BBB opening was shown by focally increased contrast enhancement on MRI. In two of five patients, sonicated and unsonicated tissue samples were compared after surgery and demonstrated increased chemotherapy concentrations in sonicated tissue. Idbaih et al.^[^ ^28^ ^]^ provided the first clinical outcome data, recorded in a series of 19 patients with recurrent glioblastoma. Patients were treated with an implanted single channel system, and the BBB was opened monthly and just before a cycle of intravenous chemotherapy. Patients with clear BBB disruption increased their survival time by about 4 months compared to patients with poor BBB disruptions.

Innovative BBB treatment studies have also been performed for Alzheimer's disease (AD). A first investigation was published with five early to moderate Alzheimer's patients using a 1024 channel helmet system.^[^ ^24^, ^30^ ^]^ The authors opened the BBB in the dorsolateral prefrontal cortex—a possible target for Alzheimer's brain stimulation therapy. Here the strategy has been to focally open the BBB to enhance the clearance of pathological beta‐amyloid deposits as has been shown in experimental animals of AD.^[^ ^31^ ^]^ Safety and functional consequences were investigated via anatomical and fMRI. In these patients the BBB could be safely, reversibly, and repeatedly opened. fMRI connectivity was transiently affected, but later restored without negative clinical consequences. In a similar study on six patients with early AD, the same technique was used to focally open BBB of the hippocampus and entorhinal cortex.^[^ ^32^ ^]^ These areas are particularly promising for dementia, but also possible targets for epilepsy and depression. Again, BBB opening was safe and no cognitive or neurological worsening was observed. Another pioneering clinical study was recently published for amyotrophic lateral sclerosis (ALS).^[^ ^33^ ^]^ This dramatic neurodegenerative disease focuses on the motor system and increasing evidence exists that brain cortical dysfunctions precede spinal cord dysfunctions. In four patients, eloquent motor cortex for hand and foot movements was first individually localized by fMRI. Using a helmet system, these functional spots were then targeted. BBB opening of the eloquent motor cortex was successful, safe, and reversible in all ALS patients. Interestingly, a further analysis of the ALS and AD BBB opening studies also provided first evidence that a novel fluid exchange system—the so called glymphatic system described in rodent brains—also exists in humans.^[^ ^34^ ^]^ Further patient studies are currently running in Parkinson's disease and on other tumor types including tumor metastases.

Current work on methodological improvements is related to the technical issues discussed for transcranial ultrasound surgery. An additional goal is the reduction of overall treatment duration with advanced real‐time monitoring and rapid electronic beam steering techniques. Specific questions researched for BBB opening are which substances work best for which the disease and how delivery of multiple doses of a substance to various focal areas of the brain can be optimized.

## 4 Brain Stimulation Therapy

**Figure 4:**
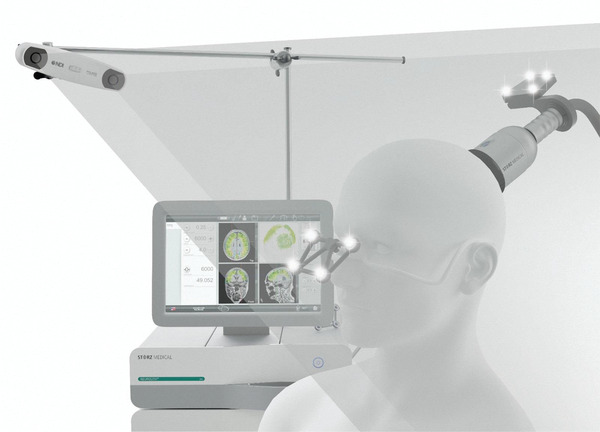
Setting with the transcranial pulse stimulation system (TPS) for individualized brain stimulation therapy.^[^ ^41^ ^]^ With TPS transcranial ultrasound can be targeted with millimeter precision to every superficial and deep area of the patient's brain.

The latest clinical development in transcranial ultrasound concerns brain stimulation therapy with focused ultrasound systems.^[^ ^35^ ^]^ This is a non‐invasive treatment of brain diseases with low energy levels, without opening of the BBB and without generating morphological changes within the brain. The basic principle is a focal modulation of neuronal activity in functionally important neuronal network spots. Short‐term effects may be either suppression or activation of neuronal activity. Long‐term effects concern neuroplastic reorganization, which may improve brain function. Within the last years, diagnostic fMRI research detected several important networks that may be clinically targeted to improve patients’ deficits, for example, the memory network, language network or motor network.^[^ ^36^ ^]^ Recent animal data have shown that these can be independently modulated by transcranial ultrasound and modulations cannot be explained by pure acoustic stimulation effects.^[^ ^37^, ^38^ ^]^ Ultrasound brain stimulation is typically performed with single channel systems equipped with neuronavigation for individualized targeting of the patient's brain. The novel technique has two major advantages compared to existing electromagnetic brain stimulation methods, for which several clinical trials exist^[^ ^39^ ^]^: 1) unprecedented precision for brain area targeting (independent of pathological conductivity changes) and 2) access to deep brain areas, which has not been possible previously. With transcranial ultrasound, non‐invasive deep brain stimulation (DBS) may become a new therapeutic option. During the last years a large variety of preclinical custom ultrasound systems have been described with considerably varying ultrasound parameters. Typically these systems use fundamental frequencies in the range of 200–1000 kHz, which are then modulated to generate ultrasound pulses with tone burst durations in the millisecond range that are applied for several minutes to a focal spot of the brain (overview in ref. ^[^ ^40^ ^]^). In addition, a first clinical system has recently been described and CE approved (**Figure** **4**). This system applies a different ultrasound technology that is based on single ultrashort ultrasound pulses (transcranial pulse stimulation [TPS]).^[^ ^41^ ^]^ The pulse durations are not in the millisecond range but the microsecond range (about 3 µs duration), and single pulses are repeated with a frequency between 4 and 8 Hz. Again, every target area is sonicated for several minutes. The focus of a single channel transducer used for brain stimulation is about 4–5 mm wide and 2–4 cm long (full width half maximum). Larger brain areas may be stimulated by moving the handheld transducer over the scalp and targeting a predefined volume on individual MR images.

There are several hypotheses concerning how low energy ultrasound for brain stimulation may change neuronal activity, although much research is still needed. A likely basis are mechanical effects on cell membranes affecting mechanosensitive ion channels and generating membrane pores. As a consequence, transmitter and humoral factor concentrations change. A recent study investigated a neuroinflammation model in a microglia cell culture.^[^ ^42^ ^]^ Low intensity pulsed ultrasound increased production of several neurotrophic factors including the neuroprotective BDNF (brain derived neurotrophic factor) and reduced neuroinflammation by suppressing harming overactivation of microglia. It is increasingly recognized that antiinflammatory effects are important to improve neurodegenerative diseases. Such ultrasound effects may contribute to memory improvement in preclinical neurodegeneration studies.^[^ ^31^, ^43^ ^]^ Another study investigated effects of ultrashort ultrasound pulses on a neuronal stem cell culture.^[^ ^44^ ^]^ Here, cell proliferation and differentiation to neurons could be enhanced. A mechanism suggested by Hameroff et al.^[^ ^45^ ^]^ is, that ultrasound directly affects cytoskeletal microtubules inside neurons and glia.

In a clinical context, trials with diagnostic ultrasound systems^[^ ^45^, ^46^ ^]^ or non‐navigated pulse stimulation^[^ ^47^ ^]^ have already been performed. However, for broad clinical application precise targeting with neuronavigation is required. Recent methodological advances now allow for accurate stimulation of small areas throughout the whole brain. Advances also enable transcranial ultrasound application all over the skull—independent from sonication windows. Sophisticated focusation and neuronavigation systems have been developed, which include real time feedback of the current stimulation focus on individual brain images. Progress has also been made to avoid brain heating and secondary stimulation maxima.^[^ ^41^ ^]^

For a typical brain stimulation study with healthy human subjects a single target area—which is important for the investigated brain function—is individually predefined and then targeted with or without neuronavigation. Changes in brain function are monitored behaviorally and via electroencephalography or fMRI. Currently, such studies investigated immediate neuromodulation effects, occurring up to about 1 h post treatment. However, very recently a first clinical study was performed and also investigated long‐term effects in 35 Alzheimer's patients.^[^ ^41^ ^]^ Using the clinically approved TPS technique, data from precise stimulation of a single small focus (primary somatosensory cortex) and from stimulating multiple areas of a cognitive network have been recorded. With TPS, the typical procedure requires recording of individual anatomical MRI data for individual definition of target areas. Depending on the disease, considerable pathological brain changes may exist and secure target definition therefore requires specific expertise. It is also possible to target brain areas based on individual functional information achieved via fMRI. For treatment, no hair shaving is required, and the patient can sit relaxed in a chair with head support. Ultrasound gel is used to couple the freely moved transducer to the skin. Every ultrasound pulse can be exactly targeted via real‐time feedback on the individual MR images. In the first ultrasound patient study, a single treatment session lasted about 45 minutes and 6 sessions were applied over 2 weeks. Five brain areas were targeted with a total ultrasound pulse count of 6000 per session at 0.2 mJ mm^−2^ per pulse. Patients’ memory performance significantly increased after treatment and improvements could be documented for a follow‐up period of 3 months. In addition, neuropsychological improvements correlated with an upregulation of the memory network in fMRI data.

Brain stimulation therapy is particularly promising for brain diseases which may be improved by neuroplastic reorganization and for those including a neuroinflammatory component. Neurodegenerative diseases (Alzheimer's, Parkinson's), stroke, multiple sclerosis and psychiatric disorders are primary candidates with several clinical studies already running. Current work on methodological improvements concerns technical optimization of DBS and optimization of clinical treatment protocols.

## 5 Safety of Transcranial Ultrasound Therapies

For broad clinical application, transcranial ultrasound has to be safe and such evidence for all three therapeutic ultrasound techniques meanwhile exists.

For ultrasound surgery, most adverse events observed in clinical studies have been temporary and mild. Gallay et al.^[^ ^48^ ^]^ report 14 events over 180 treatments. Temporary adverse events include mild degrees of: paresthesia, worsening gait instability, unsteadiness, ataxia, occipital numbness, pin sites burning, grip weakness, syncope, facial paresis, loss of taste/dysgeusia, impaired balance, dysmetria, weakness, dysarthria, dysphagia, headache, fatigue, tinnitus, disequilibrium, vestibular symptoms (nausea/vomiting/dizziness), and anxiety. Only described to persist until end of the last follow up (1–4 years later): paresthesia, worsening gait instability, facial paresis, dysmetria, weakness, disequilibrium, dysgeusia. The most common events are paresthesia and gait disturbance/ataxia.^[^ ^5^, ^11^ ^]^ Even in geriatric patients the procedure was well‐tolerated.^[^ ^13^ ^]^

For BBB opening, energy levels of transcranial ultrasound are much lower than with ultrasound surgery. A large amount of preclinical and recent clinical studies suggest that BBB opening can be done safely. Potential adverse effects include microhemorrhage, overt hemorrhage from vascular rupture, ischemia from vascular constriction, cerebral edema, inflammation and direct cellular injury from heat or mechanical forces. In safety profile studies with animals, petechiae due to vascular damage has most commonly been reported for repeated BBB opening.^[^ ^6^, ^49^ ^]^ A sterile inflammatory brain response has also been reported.^[^ ^50^ ^]^ The occurrence of adverse effects depends on a large variety of factors including microbubble type, microbubble dosage, and ultrasound parameters. The clinical patient studies reported mostly mild to moderate adverse effects: headache, vagal responses, musculosceletal pain, scalp rash, fatigue, transient facial palsy, brain edema, and asymptomatic and transient radiological hyper‐ or hypointensities. Some of these may be related to the stereotactic framing procedure. Specific framing events have been reported as pain, edema and bruising. The largest currently published study investigated 19 patients with glioblastoma and carboplatin infusion following BBB opening^[^ ^28^ ^]^ and additionally reported hematologic changes. This study found fatigue (42%) as the most common adverse effect, followed by headache and thrombocytopenia (26%). The most recent patient study reported no treatment related adverse effects (AD patients).^[^ ^32^ ^]^

Brain stimulation applies the lowest energy levels of all transcranial ultrasound technologies since the technique tries to avoid BBB opening. In previous animal studies, partly testing high energy dosing, the following local events have been described on a very rare basis: bleeding, possible cell damage, temperature rise up to 3 °C, and BBB opening.^[^ ^40^ ^]^

There is a particular risk for such events when the ultrasound technique allows intensity hotspots due to unintended standing waves and focusing effects of the skull.^[^ ^51^ ^]^ However, recent technologies have been considerably improved and a novel pulsed stimulation approach has been introduced, which can avoid these dangers (TPS).^[^ ^41^ ^]^ Extending the previous animal data, the TPS study is also the first to report on minor transient events associated with ultrasound brain stimulation in humans. The authors found rare events of headache (4% of participants), mood deterioration (3%), pain (8%), and painless pressure sensations (17%) in their patient population. In total, 45 subjects participated in the comprehensive investigation (10 healthy subjects, 35 Alzheimer's patients). A second study reporting minor adverse events in humans was recently published with 24 healthy participants.^[^ ^52^ ^]^ Here, transducer‐related sensations were described during active stimulation (10/24 participants) and during sham (7/24) and classified as “pulsing,” “buzzing,” “pressure,” and “warm.” Following these, a third recent report assembled data from 64 subjects over 7 experiments.^[^ ^53^ ^]^ Events were classified as possibly/probably related and unlikely/unrelated to ultrasound stimulation. For the first class, neck pain, difficulty paying attention, muscles twitches and anxiety were described (7/64). For the second class, severe unusual feelings/attitudes/emotions (1), sleepiness (15), headache (4), itchiness (5), tooth pain (1), and forgetfulness (4) were described. Other human studies did not report any adverse events with ultrasound brain stimulation.^[^ ^54^, ^55^, ^56^, ^57^ ^]^

## 6 Conclusion

The last years have seen a fascinating rally to develop novel concepts for ultrasound brain therapy. Now, highly focused ultrasound beams allow non‐invasive surgery, focal transmission of therapeutic drugs or genes, and therapeutic modulation of neuronal networks for various brain diseases. Many of these techniques represent novel add‐on options, allowing us to continue already established therapies. Recent patient data show, that the transcranial ultrasound innovations are safe and ready for broad clinical application.

## Conflict of Interest

The authors declare no conflict of interest.
